# Cognitive Performance of Göttingen Minipigs Is Affected by Diet in a Spatial Hole-Board Discrimination Test

**DOI:** 10.1371/journal.pone.0079429

**Published:** 2013-11-05

**Authors:** Annika Maria Juul Haagensen, Anders Bue Klein, Anders Ettrup, Lindsay R. Matthews, Dorte Bratbo Sørensen

**Affiliations:** 1 Department of Veterinary Disease Biology, Section of Experimental Animal Models, Faculty of Health and Medical Sciences, University of Copenhagen, Frederiksberg, Denmark; 2 Department of Drug Design and Pharmacology, Faculty of Health and Medical Sciences, University of Copenhagen, Copenhagen, Denmark; 3 Neurobiology Research Unit, Copenhagen University Hospital, Rigshospitalet, Copenhagen, Denmark; 4 Lindsay R Matthews & Associates Research International, Scerne Di Pineto, Italy; 5 Psychology Department, The University of Auckland, Auckland, New Zealand; Centre national de la recherche scientifique, France

## Abstract

Consumption of a high energy diet, containing high amounts of saturated fat and refined sugar has been associated with impairment of cognitive function in rodents and humans. We sought to contrast the effect of a high fat/cholesterol, low carbohydrate diet and a low fat, high carbohydrate/sucrose diet, relative to a standard low fat, high carbohydrate minipig diet on spatial cognition with regards to working memory and reference memory in 24 male Göttingen minipigs performing in a spatial hole-board discrimination test. We found that both working memory and reference memory were impaired by both diets relative to a standard minipig diet high in carbohydrate, low in fat and sugar. The different diets did not impact levels of brain-derived neurotrophic factor in brain tissue and neither did they affect circulatory inflammation measured by concentrations of C-reactive protein and haptoglobin in serum. However, higher levels of triglycerides were observed for minipigs fed the diets with high fat/cholesterol, low carbohydrate and low fat, high carbohydrate/sucrose compared to minipigs fed a standard minipig diet. This might explain the observed impairments in spatial cognition. These findings suggest that high dietary intake of both fat and sugar may impair spatial cognition which could be relevant for mental functioning in humans.

## Introduction

The typical diet consumed by people in Western countries is high in energy, containing high amounts of refined sugar and saturated fat. Other than playing a well-known role in obesity and other life-style-related diseases like type 2 diabetes and cardio-vascular diseases, there is also increasing evidence from human studies that consumption of a high energy diet can have negative implications on cognitive function [1-4]. High energy diets might also play a role in the development of Alzheimer’s disease [5]. Rodent studies have shown that diets high in fat and sugar result in both spatial and non-spatial impaired hippocampus-related learning and memory [6,7]. Furthermore, impaired spatial cognition has been reported after consumption of high levels of saturated fat in both rats and mice [8-11]. Jurdak and colleagues found that excess sucrose intake, but not excess saturated fat intake impaired spatial learning and memory in young obese rats [12]. Other rodent studies also indicate a negative effect of sucrose or fructose on cognition [13-16], and specific impairment of spatial cognition related to feeding of a high energy diet [17] or a high fructose intake [15,16] have been reported independent of the onset of obesity. In contrast to rodent studies, more inconsistencies are reported in human studies; with children, no effect on cognitive performance with a high intake of sugar (sucrose; 103-120g/day) was found in one study [18] while another reported impaired cognitive function with high intake of refined carbohydrates (rice, white bread and white flour, sugar, food items containing sugar and sugar-sweetened beverages; 128-285g/day) [19]. Thus, primarily evidenced from rodent studies, high intake of fat and sugar seem to have a negative effect on especially spatial cognition, whether it is consumed independently or in combination. 

Brain-derived neurotrophic factor (BDNF) is a growth factor in the neurotrophin family, which is expressed throughout the brain, with high expression in the cortex and hippocampus. The hippocampus is a brain region highly involved in spatial learning and memory [20]. Studies show that BDNF is playing a role in learning and memory processes [6,21], and regulation of BDNF has been linked to dietary factors. For example, studies in rats and mice have reported a reduction in BDNF levels after feeding with high energy diets [11,22]. 

Increased levels of circulating C-reactive protein (CRP) have been correlated with changes in brain metabolites indicating early brain vulnerability [23], and serum CRP levels has also been shown to be elevated in patients with mild cognitive impairment, a prodromal stage of Alzheimer’s disease [24]. In a 6-year follow-up study on 65 healthy aging humans, cognitive impairment was investigated with regards to serum concentrations of inflammatory proteins. In this study it was concluded that high concentrations of haptoglobin and CRP might be indicators of impaired cognitive performance [25]. Increased concentrations of CRP and haptoglobin have been associated with diets high in sucrose as well as high glycaemic load [26,27]. Hence, serum elevation of inflammatory markers such as CRP and haptoglobin might be caused by dietary factors and might lead to impaired cognitive performance. 

Studies in rats and mice have led to associations between diet-induced high blood levels of triglycerides (TG) and impaired cognition, related to both consumption of a high sugar- or high fat diet [12,15,28]. A few human studies have suggested that elevated TG levels might lead to delirium [29,30] and might also be associated with poor cognitive performance in persons with type 2 diabetes [31]. 

The pig has become a highly-valued animal model when studying implications of a variety of human diseases. The pig as a model animal has a high translational value due to its close similarity to humans; pigs have a large gyrencephalic brain, allowing for the possibility to perform high-resolution brain imaging, also, pigs are omnivorous and have a digestive system functioning very similarly to that of humans. The use of the pig as a model in neuroscience is increasing [32,33] and the minipig has become especially important, due to its commercial availability and relatively small size, though still allowing for easy collection of body fluids and tissue samples. Also, much is known about the minipig’s physiology, anatomy and metabolism [34].

In 2009, Arts and colleagues [35] adjusted the rodent spatial hole-board discrimination task for pigs, enabling the measurement of working- and reference memory. This test has so far been used to investigate possible spatial learning and memory impairments in young pigs subjected to mixing stress, which was found not to affect cognitive function [35], and in piglets born with a low birth weight, who were found to be transiently impaired in reversal learning [36]. With minor adjustments in the setup, performance of juvenile Göttingen minipigs in a spatial hole-board discrimination task has been investigated regarding possible use of this test within pre-clinical toxicity testing, showing high potential for implementation [37,38]. Studies of dietary effects on cognitive function in pigs are scarce, but one study has shown positive effect of a nutritional supplementation (sialic acid) on learning and memory in piglets [39].

A relatively large amount of data from rodent studies has demonstrated dietary effects on cognition. However, the situation is less evidenced in humans hence the importance to evaluate such possible effects in a relevant alternate animal model like the minipig. We contrasted the effect of a high fat/cholesterol, low carbohydrate diet with a low fat, high carbohydrate/sucrose diet and a standard minipig diet high in carbohydrate, low in fat and sugar. Possible effects on working memory and reference memory performance were assessed in 24 male Göttingen minipigs performing a spatial hole-board discrimination test. We also investigated the relationship between cognitive performance and serum levels of TG, the inflammatory markers CRP and haptoglobin in serum and BDNF levels in plasma and brain tissue. 

## Methods

### Ethics statement

Animals were treated in accordance with the Animal Experimentation Act of Denmark, which is in accordance with the Council of Europe Convention ETS 123. The study was approved by the Danish Animal Experimentation Board under the Ministry of Food, Agriculture and Fisheries of Denmark (Permit Number: 561-1434). 

### Animals and housing

24 male Göttingen specific-pathogen-free (SPF) minipigs (Ellegaard Göttingen minipigs A/S, Denmark) were the subjects of this study with 8 minipigs per diet treatment (LFHC: low fat, high carbohydrate; LFHS: low fat, high carbohydrate/sucrose; HFLC: high fat/cholesterol, low carbohydrate). See Diets section for detailed information. The minipigs arrived at age 6 weeks and were allowed two weeks of acclimatisation before study start. At age 21 weeks, the study was ended and minipigs were euthanized. The study was carried out in two replicates (A and B) of 12 minipigs. The minipigs were assigned to one of three sub-groups (diet treatments: LFHC, LFHS, HFLC) of 4 minipigs balanced for genetic and social relatedness. Hence, all minipigs originated from different parents/litters and if housed together before their arrival, these animals were assigned to different sub-groups. The subgroups were housed in pens measuring 3m x 3m, allowing social contact between groups housed in adjacent pens. All minipigs were individually numbered with a marker pen on forehead, flanks and back. The pens were cleaned and provided with fresh wood shavings, straw and hay every morning. Additionally, each pen was equipped with a heat lamp (for minipigs aged 6-12 weeks). The animal room was provided with, an 8-/16-hour light-dark cycle (lights on from 7 AM to 3 PM) as well as natural lighting from three skylights and filtered air at a temperature of 22ᵒC ± 3ᵒC. All tests were performed during daylight hours. The study was conducted at the Laboratory Animal Facility, Faculty of Health and Medical Sciences, University of Copenhagen, Denmark between March and November. 

### Diets

From 6 to 8 weeks of age, during acclimatisation, the minipigs were fed three times daily with their regular diet, which had been fed to the minipigs post weaning by the breeder (Ellegaard Göttingen minipigs A/S). As the regular diet of the two batches of minipigs (A and B) were slightly different due to a change in the use of diets at the breeder, batch A received pre-test diet A (Standard minipig, Piglet diet 10kGy, Special Diets Services, UK) and batch B received pre-test diet B (Standard minipig diet, special quality control, Special Diets Services, UK) (Table 1). From the start of the experimental period, at 8 weeks of age, animals in each treatment received the assigned test diet three times daily (Table 1). The diets were cereal based raw material diets. Food rations were calculated and adjusted weekly during the study period to meet the normal weight curve of the animals according to their age based on the following equation: Metabolisable energy = 1744 kJ x Body weight^0.52^ [40]. Minipigs were group fed in their home pens, where the feed was equally distributed between four bowls. A standard minipig diet (Standard minipig diet, Special Diets Services, UK) served as the control diet. This is a low fat, high carbohydrate diet (LFHC). For the low fat high carbohydrate/sucrose diet (LFHS), the standard minipig diet was used and supplemented with Sucrose (Catalog no. 84100, purity ≥99.0%, crystal sugar, Sigma-Aldrich, Denmark A/S). A modified standard minipig diet (Standard minipig diet 17% lard/2% cholesterol, Special Diets Services, UK) was used for the high fat/cholesterol, low carbohydrate diet (HFLC). To ensure the same exposure to all the nutrients apart from an increased exposure to fat and energy HFLC minipigs were fed 120g for every 100g fed to the minipigs in the LFHC group. The difference in energy intake per day (kJ) were calculated, between these two groups of minipigs, and the amount of added sucrose fed to minipigs in the LFHS group was matched to meet the metabolisable energy (MJ/kg) of the HFLC diet and hence also adjusted weekly (79g-115g sucrose/pig/day). The sucrose was mixed with the standard minipig diet ensuring equally distribution between the four feeding bowls. Water was provided *ad libitum* in all three groups. In behavioural tests where positive reinforcement was used, minipigs received a food reward matching their respective diet i.e. in the spatial hole-board discrimination test control minipigs were rewarded with pellets (2 pellets x 4 bowls ^≈^ 2 g) from the standard minipig diet, minipigs from the high sugar treatment were rewarded with small pieces of sucrose (1/4 cube sugar x 4 bowls ^≈^ 2g) and minipigs on the high fat/cholesterol diet were rewarded with small pieces (1 piece x 4 bowls ^≈^ 2g) of lard (a by-product of melted lard, mainly consisting of small pieces of connective tissue). These rewards were chosen to match the respective dietary treatments as closely as possible and to ensure a high motivation of minipigs from all diet groups for obtaining the rewards. Ensuring a high motivation in all three groups was not possible in LFHS and HFLC minipigs using only standard pellets as the animals did not want to work for this reward. Also, standardising the reward of all three groups using either lard or sugar could have confounded the dietary treatments. On test days, minipigs received 50% of their normal food ration at morning and afternoon feedings, before the two daily tests. The remaining 50% was provided with the last feed of the day, when testing was completed. 

**Table 1:** Pre-test diets and experimental diets.

Pre-test diet A (age 6-7 weeks)		LFHC/LFHS/HFLC	
**Crude protein (kcal)**		14.20%	
**Crude fibre (kcal)**		12.70%	
**Crude fat (kcal)**		4.50%	
**Starch (kcal)**		22.50%	
**Sugar (kcal)**		9.20%	
Pre-test diet B (age 6-7 weeks)			
**Crude protein (kcal)**		13.00%	
**Crude fibre (kcal)**		14.50%	
**Crude fat (kcal)**		2.10%	
**Starch (kcal)**		27.10%	
**Sugar (kcal)**		5.50%	
Experimental diets (age 8-21weeks)	**LFHC**	**LFHS + sucrose**	**HFLC**
**Crude protein (kcal)**	13.03%	10.86%	10.76%
**Crude fibre (kcal)**	14.52%	12.10%	11.20%
**Crude fat (kcal)**	2.13%	1.77%	17.51%
**Starch (kcal)**	27.12%	22.60%	21.83%
**Sugar (kcal)**	5.54%	21.54%	4.90%
AFE (experimental diets)			
**Crude protein (kcal)**	18.60%	14.34%	12.00%
**Crude fat (kcal)**	6.80%	5.25%	42.00%
**Carbohydrate (kcal)**	74.60%	80.40%	46.00%
ME (experimental diets)			
**Total (MJ/kg)**	10.98	11.98	11.98

Pre-test diet A was fed to the first batch of minipigs (A) and pre-test diet B was fed to the second batch of minipigs (B) during the two weeks of acclimatisation before the test diets were applied. Low fat, high carbohydrate (LFHC); low fat, high carbohydrate/sucrose (LFHS); high fat/cholesterol, low carbohydrate (HFLC); Atwater fuel energy (AFE); Metabolisable energy (ME).

### Spatial hole-board discrimination test

#### Test area and apparatus

At the age of 15-16 weeks and 18-19 weeks, minipigs were subjected to a spatial hole-board discrimination test. The test consisted of: an acquisition phase (A) of 10 days, which was followed by a retention interval of 9 days. Hereafter, minipigs were tested in a memory phase (M) for 4 consecutive days, which was followed by a reversal learning phase (R) of 4 consecutive days. Sixteen plastic feeding bowls (diameter 11.5cm, height 8cm) were taped to the floor in an arena (3m x 3m) with solid walls (1.2m). The bowls were placed in a square (4 x 4 bowls) with equal amount of space between adjacent bowls and walls. Each bowl was provided with a perforated fixed inner bowl, separating accessible food from inaccessible food. To prevent olfactory guidance, all bowls contained inaccessible food according to the respective reward diets. Four out of the sixteen bowls were baited with a food reward accessible to the minipig. The minipigs were randomly, but evenly distributed between the three diets, assigned one of two configurations of four baited bowls. This configuration was fixed during the A phase and M phase and reversed during the R phase. Minipigs were individually led into the arena through one of two manually operated guillotine doors, each provided with an adjacent waiting area (startbox) measuring 1m x 0.8m. The operator was located at a fixed position by the side of the arena visible to the minipigs. Similarly, an observer who recorded the performance of the minipigs stood by the side of the arena. Visual cues (black dots on white background, exchanging vertical stripes of black and white and a black sheet) were placed on the walls inside the arena, except for the wall of the entrances. The test area was placed inside the animal room, hence, minipigs were able to hear and smell but not see each other, during testing.

#### Training and testing

From day one the minipigs were pre-exposed to the arena food bowls in their home pen, learning that they contained food. In addition to the daily routines, the minipigs were socialized towards humans three times a week. Following procedures were executed: Initiating voluntary physical contact between a known human handler and each of the minipigs as well as training simple handling procedures like going in and out of the home pen and getting picked up. The socialization and training was done by the use of positive reinforcement training during periods when no other behavioural testing was conducted. At the age of 14 weeks minipigs were pre-exposed to the test area of the spatial hole-board discrimination test for one week. Pre-exposure started out in groups of four minipigs (pen mates) which were gradually reduced [1,2,4] to single minipigs exploring the setup. In the beginning, a mixture of pellets and reward food was scattered both around the floor of the arena, in the startboxes and in each of the bowls. Minipigs then stayed in the test area until all the food was eaten or a maximum of 20 minutes had past. Hereafter, accessible food was gradually reduced, ending with half the food bowls being randomly baited with reward food. For the last two days of pre-exposure, minipigs were singly and twice a day habituated to the startboxes for 1 minute. 

When tested, a minipig was let into the arena after staying 1 minute in the startbox, and they were allowed to locate and collect food rewards from the four baited bowls for a maximum of 10 minutes. A visit to a bowl was recorded whenever the minipig touched a bowl with its snout or placed the snout above the bowl [41]. For each trial, time to complete the trial (trial duration), number of visits and revisits to baited bowls as well as non-baited bowls, order of visits and number of food rewards eaten were recorded. By the end of a trial the door of the startbox was opened and when returning the minipig received a food reward. Between trials, the floor of the arena and startbox was dry- mopped, bowls were cleaned with a wet towel and food was replaced or exchanged. The minipigs were tested in a random order and went through two daily trials with an inter-trial interval of 3-4 hours. The entrances were alternated between morning and afternoon testing.

### Blood samples and brain tissue samples

Blood samples were collected in fasted minipigs at 3 time points during the study period; age 8 weeks (baseline), age 13 weeks and age 21 weeks (euthanasia). Venous blood was collected from the cranial vena cava in serum- and K_2_EDTA tubes (BD Vacutainer^TM^) for CRP, haptoglobin, TG and BDNF respectively. Samples were centrifuged (5 min., 3500 rpm) and stored at -80ᵒC until further analysis. The minipigs were awake during blood sampling, except on the day of euthanasia, where they were sedated (intramuscularly, with a mixture of 1 mg/kg midazolam (Midazolam Hameln 5 mg/ml, Hameln Pharmaceuticals gmbh, Germany) and 10 mg/kg ketamine (Ketamine Vet 100 mg/ml, Intervet, Denmark)). Subsequently, an intravenous access was provided in all pigs and 1-2 mg/kg propofol (Rapinovet Vet 10 mg/ml, Schering-Plough Animal Health, Denmark) was given if needed. Minipigs were euthanized by an intravenous overdose (150 mg/kg) of pentobarbital (Pentobarbital 200mg/ml, Glostrup Apotek, Denmark), they were decapitated and the brain was quickly removed for immediate dissection. Brain tissue samples were obtained from frontal cortex (cranio-medial part of tissue excised 2 cm from the rostral tip of the cerebrum) and hippocampus (medial part excised after the hippocampus was dissected bluntly in its full length), snap frozen in liquid nitrogen and then stored at -80ᵒC until further analysis of BDNF levels.

BDNF levels in plasma samples were analysed using a commercially available immunoassay kit BDNF E_max_^®^ Immunoassay System (Promega, Sweden). Tissue samples of frontal cortex and hippocampus were homogenized in RIPA buffer added a protease inhibitor; 2 nM Na_3_VO_4_ and 48nM NaF, sonicated for 3x5 sec. on ice followed by centrifugation (4ᵒC, 10000 rpm for 10 min.). The supernatant was stored at -80ᵒC until protein concentrations were measured by the modified Lowry method (DC Protein Assay, Bio-Rad, Denmark). BDNF was then measured by enzyme-linked immunosorbent assay (ELISA) method (Promega, Sweden) and absorbance measured on an ELISA reader (MicroPlate Reader 550, Bio-Rad, Denmark). The BDNF standards contained in the kit is within the range 7.82-500 pg/ml. However, samples were diluted at least 1:2 in sample buffer resulting in a detection limit of ^≈^ 16 pg/ml [42]. The BDNF ELISA assay has previously been validated for pigs [42].

Serum concentrations of CRP and haptoglobin were analysed on an Adiva 1800 Chemistry system (Siemens), with a haptoglobin reagent from TriDelta and a CRP reagent from Randox. TG measurements were done on a Horiba ABX Pentra 400 Chemestry Analyser (Horiba ABX, France).

### Data analysis

GraphPad Prism 5.02 was used for statistical calculations. Working memory was defined as (number of food rewarded visits/(number of visits and revisits to the baited set of bowls)) and reference memory defined as ((number of visits and revisits to the baited set of bowls)/number of visits and revisits to all bowls) [35]. Working memory score was calculated as the number of food rewarded visits (RewVis) relative to the number of visits and revisits to the baited bowls (VisBB, RVisBB): Working memory score = RewVis/(VisBB+RVisBB). Reference memory score was calculated as the number of visits and revisits to baited bowls (VisBB, RVisBB) relative to the total number (N) of visits (including revisits) to all bowls: Reference memory score = (VisBB+RVisBB)/N. Data were averaged over blocks of four trials when calculating working memory scores, reference memory scores and trial duration for the acquisition phase, memory phase and reversal learning phase. Any missing data points/trials (A phase: LFHC (0.625%), HFLC (1.25%), LFHS (25%); M phase: LFHC (0%), HFLC (12.5%), LFHS (25%); R phase: LFHC (18.75%), HFLC (0%), LFHS (25%)) were dealt with by Median Imputation; replacing by the median of all known values of that attribute in the class where the instance with the missing feature belongs [43]. Differences, in working memory, reference memory and trial duration, within and between phases were assessed by within subject analysis of variance (ANOVA) with the repeated measures factor trial blocks, followed by a Tukey’s post-test for effect of trial block and the between subjects factor diets, followed by a Bonferroni post-test for effect of diet. Analyses of differences in BDNF, CRP, haptoglobin and TG levels as well as body weight were carried out by one-way analysis of variance (ANOVA) and unpaired t-test. A two tailed Spearman rank correlation test was used to assess any correlation between body weight and performance in the spatial hole-board discrimination test (working memory scores, reference memory scores and trial duration). 

## Results

**Figure 1:**
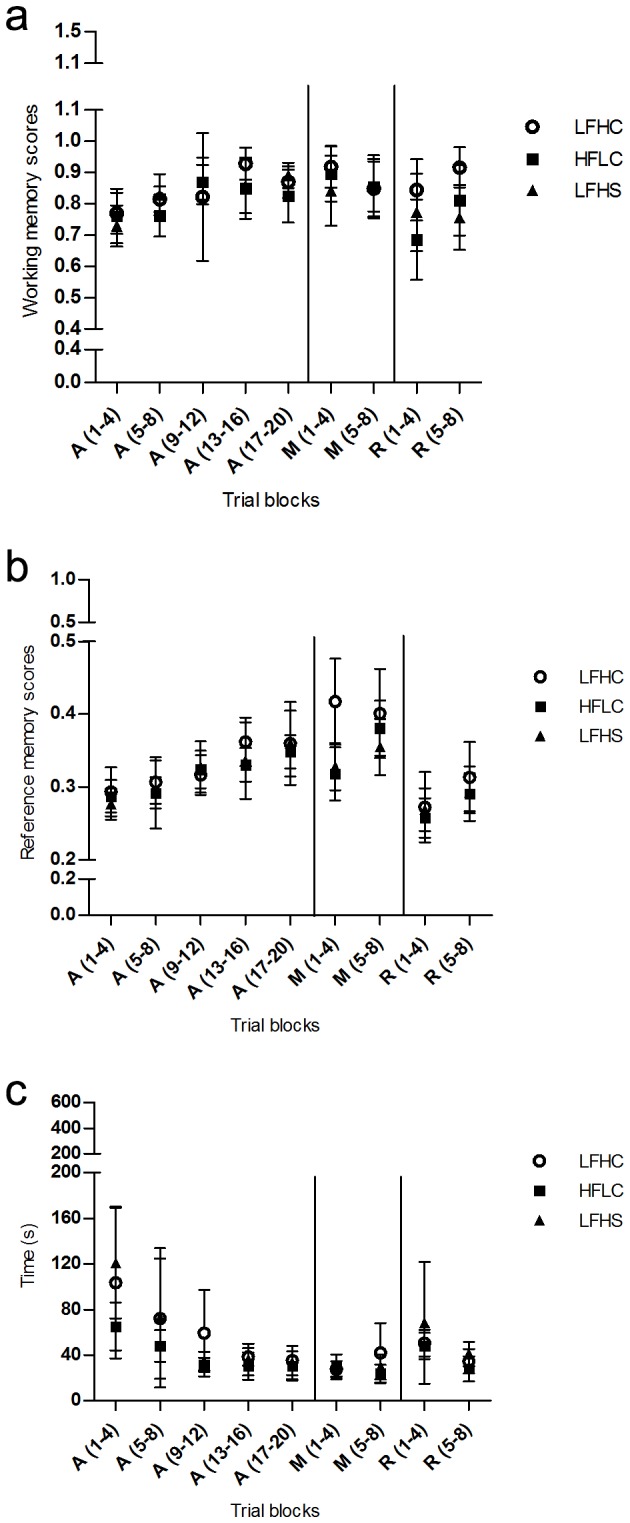
a-c. Performance of minipigs in the spatial hole-board discrimination test. Data are presented as trial blocks of means of four trials ±SD for the acquisition phase (A), the memory phase (M) and the reversal learning phase (R) where a = working memory scores, b = reference memory scores and c = trial duration (time, sec.). Low fat, high carbohydrate diet (LFHC); high fat/cholesterol, low carbohydrate diet (HFLC); low fat, high carbohydrate/sucrose diet (LFHS).

**Figure 2:**
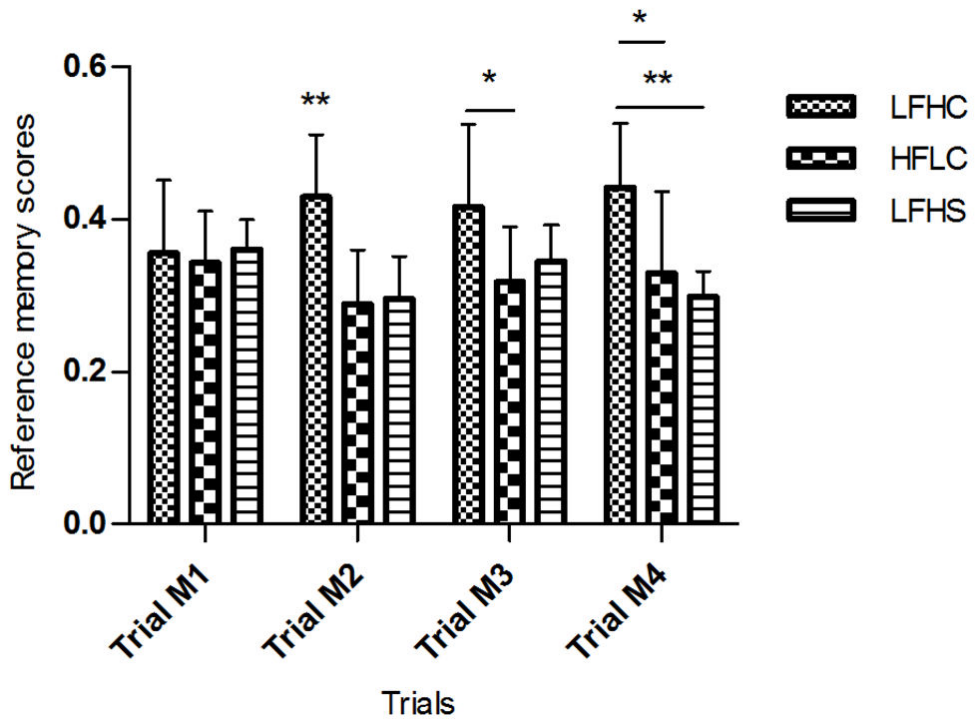
Reference memory performance of minipigs in the spatial hole-board discrimination test. Data are presented as mean scores for the first four trials of the memory phase (M) for low fat, high carbohydrate diet (LFHC); high fat/cholesterol, low carbohydrate diet (HFLC) and low fat, high carbohydrate/sucrose diet (LFHS). * p < 0.05, ** p < 0.01.

The minipigs learned to perform in the spatial hole-board discrimination test consisting of three phases; acquisition, memory and reversal learning wherein scores of working memory, reference memory and trial duration was calculated. Both trial block and diet had an effect on performance of the minipigs (Table 2, Figure 1a-c): Minipigs significantly improved working memory scores (LFHC: F_4,28_ = 2.867, p < 0.05; LFHS: F_4,28_ = 6.970, p < 0.001; HFLC: F_4,28_ = 3.647, p < 0.05) and reference memory scores (LFHC: F_4,28_ = 7.969, p < 0.001; LFHS: F_4,28_ = 6.684, p < 0.001; HFLC: F_4,28_ = 5.924, p < 0.01) over time during the A phase with no effect of diet. Trial duration decreased during the A phase (LFHC: F_4,28_ = 4.182, p < 0.01; LFHS: F_4,28_ = 10.77, p < 0.0001; HFLC: F_4,28_ = 9.533, p < 0.0001) and an effect of diet was found (F_2,168_ = 4.763, p < 0.05). Further analysis showed that this diet effect was only present during the first trial block A(1-4) where LFHS minipigs took a significantly longer time to complete a trial (p < 0.001) compared to HFLC minipigs. An effect of trial block was found for the M phase for working memory scores (LFHC: F_6,42_ = 2.908, p < 0.05; LFHS: F_6,42_ = 3.387, p < 0.01; HFLC: F_6,42_ = 3.443, p < 0.01), reference memory scores (LFHC: F_6,42_ = 11.000, p < 0.001; LFHS: F_6,42_ = 5.505, p < 0.001; HFLC: F_6,42_ = 8.359, p < 0.001) and trial duration (LFHC: F_6,42_ = 4.634, p < 0.01; LFHS: F_6,42_ = 10.830, p < 0.001; HFLC: F_6,42_ = 11.260, p < 0.001). Diet had no effect on working memory performance or trial duration during the M phase. In contrast, an effect of diet was found on reference memory scores (F_2,168_ = 7.710, p < 0.01) where LFHC minipigs performed significantly better (p < 0.001) than LFHS and HFLC minipigs during the first trial block; M (1-4). This difference was found for the last three trials of the block (Figure 2). Some effect of trial block was found in the R phase for working memory scores (LFHC: NS; LFHS: NS; HFLC: F_8,56_ = 4.209, p < 0.001), reference memory scores (LFHC: F_8,56_ = 9.768, p < 0.001; LFHS: F_8,56_ = 7.179, p < 0.001; HFLC: F_8,56_ = 10.340, p < 0.001) and trial duration (LFHC: F_8,56_ = 4.646, p < 0.001; LFHS: F_8,56_ = 6.730, p < 0.001; HFLC: F_8,56_ = 9.759, p < 0.001). No effect of diet was found for reference memory performance or trial duration in the R phase. In contrast, working memory scores were different between diets (F_2,168_ = 4.091, p < 0.05) with LFHC minipigs performing significantly better (p < 0.01) than HFLC and LFHS minipigs in the first trial block; R(1-4) and second trial block; R(5-8), respectively. We found no fixed search pattern for any of the minipigs when looking at the order in which the bowls were visited.

**Table 2:** Trial block effect on performance of minipigs in the spatial hole-board discrimination test.

Trial block comparisons		LFHC diet			HFLC			LFHS	
	**WM**	**RM**	**T**	**WM**	**RM**	**T**	**WM**	**RM**	**T**
**A (1-4) vs. A (5-8)**	NS	NS	NS	NS	NS	NS	p<0.05	NS	p<0.05
**A (1-4) vs. A (9-12)**	NS	NS	NS	p<0.05	NS	p<0.001	p<0.01	NS	p<0.001
**A (1-4) vs. A (13-16)**	p<0.05	p<0.01	p<0.05	NS	NS	p<0.001	p<0.01	p<0.05	p<0.001
**A (1-4) vs. A (17-20)**	NS	p<0.01	p<0.05	NS	p<0.01	p<0.001	p<0.001	p<0.001	p<0.001
**A (1-4) vs. M (1-4)**	p<0.05	p<0.001	p<0.01	p<0.05	NS	p<0.001	NS	NS	p<0.001
**A (1-4) vs. M (5-8)**	NS	p<0.001	p<0.05	NS	p<0.001	p<0.001	p<0.05	p<0.01	p<0.001
**A (1-4) vs. R (1-4)**	NS	NS	p<0.05	NS	NS	NS	NS	NS	NS
**A (1-4) vs. R (5-8)**	NS	NS	p<0.01	NS	NS	p<0.001	NS	NS	p<0.001
**A (5-8) vs. A (9-12)**	NS	NS	NS	NS	NS	NS	NS	NS	NS
**A (5-8) vs. A (13-16)**	NS	p<0.05	NS	NS	NS	NS	NS	NS	NS
**A (5-8) vs. A (17-20)**	NS	p<0.05	NS	NS	p<0.01	NS	NS	p<0.01	NS
**A (5-8) vs. M (1-4)**	NS	p<0.001	NS	NS	NS	NS	NS	NS	NS
**A (5-8) vs. M (5-8)**	NS	p<0.001	NS	NS	p<0.001	p<0.01	NS	p<0.05	NS
**A (5-8) vs. R (1-4)**	NS	NS	NS	NS	NS	NS	NS	NS	NS
**A (5-8) vs. R (5-8)**	NS	NS	NS	NS	NS	p<0.05	NS	NS	NS
**A (9-12) vs. A (13-16)**	NS	NS	NS	NS	NS	NS	NS	NS	NS
**A (9-12) vs. A (17-20)**	NS	NS	NS	NS	NS	NS	NS	NS	NS
**A (9-12) vs. M (1-4)**	NS	p<0.001	NS	NS	NS	NS	NS	NS	NS
**A (9-12) vs. M (5-8)**	NS	p<0.001	NS	NS	p<0.05	NS	NS	NS	NS
**A (9-12) vs. R (1-4)**	NS	NS	NS	p<0.01	p<0.01	NS	NS	p<0.05	NS
**A (9-12) vs. R (5-8)**	NS	NS	NS	NS	NS	NS	NS	NS	NS
**A (13-16) vs. A (17-20)**	NS	NS	NS	NS	NS	NS	NS	NS	NS
**A (13-16) vs. M (1-4)**	NS	NS	NS	NS	NS	NS	NS	NS	NS
**A (13-16) vs. M (5-8)**	NS	NS	NS	NS	p<0.05	NS	NS	NS	NS
**A (13-16) vs. R (1-4)**	NS	NS	NS	p<0.05	p<0.01	NS	NS	p<0.05	NS
**A (13-16) vs. R (5-8)**	NS	NS	NS	NS	NS	NS	NS	NS	NS
**A (17-20) vs. M (1-4)**	NS	NS	NS	NS	NS	NS	NS	NS	NS
**A (17-20) vs. M (5-8)**	NS	NS	NS	NS	NS	NS	NS	NS	NS
**A (17-20) vs. R (1-4)**	NS	NS	NS	NS	p<0.001	NS	NS	p<0.001	NS
**A (17-20) vs. R (5-8)**	NS	NS	NS	NS	p<0.05	NS	NS	p<0.05	NS
**M (1-4) vs. M (5-8)**	NS	NS	NS	NS	p<0.01	NS	NS	NS	NS
**M (1-4) vs. R (1-4)**	NS	NS	NS	p<0.001	p<0.05	NS	NS	p<0.05	NS
**M (1-4) vs. R (5-8)**	NS	NS	NS	NS	NS	NS	NS	NS	NS
**M (5-8) vs. R (1-4)**	NS	NS	NS	p<0.05	p<0.001	p<0.01	NS	p<0.001	NS
**M (5-8) vs. R (5-8)**	NS	NS	NS	NS	p<0.001	NS	NS	p<0.05	NS
**R (1-4) vs. R (5-8)**	NS	NS	NS	NS	NS	p<0.05	NS	NS	NS

Low fat, high carbohydrate diet (LFHC); high fat, low carbohydrate diet (HFLC); low fat, high carbohydrate/sucrose diet (LFHS); working memory (WM); reference memory (RM); trial duration (T); acquisition phase (A); memory phase (M); reversal learning phase (R); non-significant (NS).

Two LFHS minipigs did not habituate to the arena and were therefore excluded from testing. Also, one LFHC minipig stopped working in the arena by the end of the M phase, and was excluded from further testing. 

**Figure 3:**
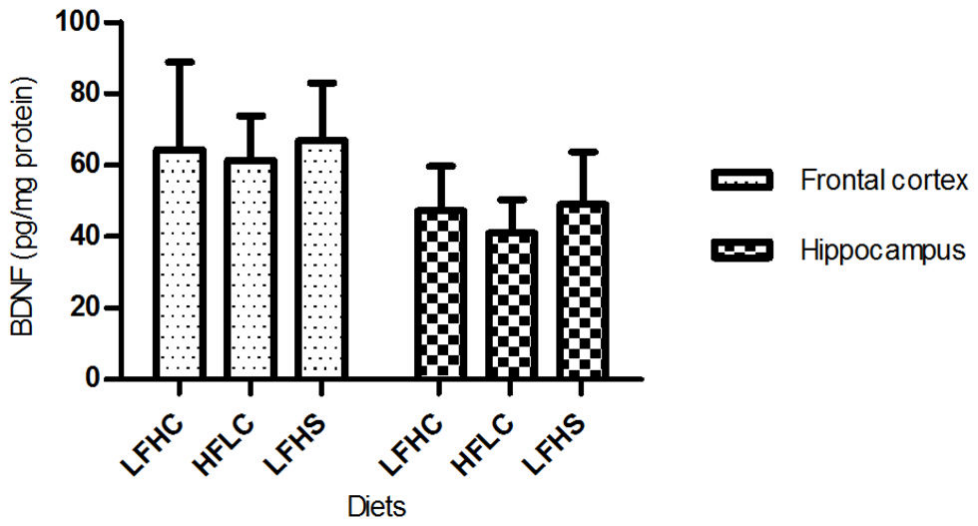
Brain-derived neurotrophic factor (BDNF) in tissue from frontal cortex and hippocampus. BDNF levels (pg/mg protein) are presented as means ±SD for minipigs fed low fat, high carbohydrate diet (LFHC); high fat/cholesterol, low carbohydrate diet (HFLC); low fat, high carbohydrate/sucrose diet (LFHS).

**Figure 4:**
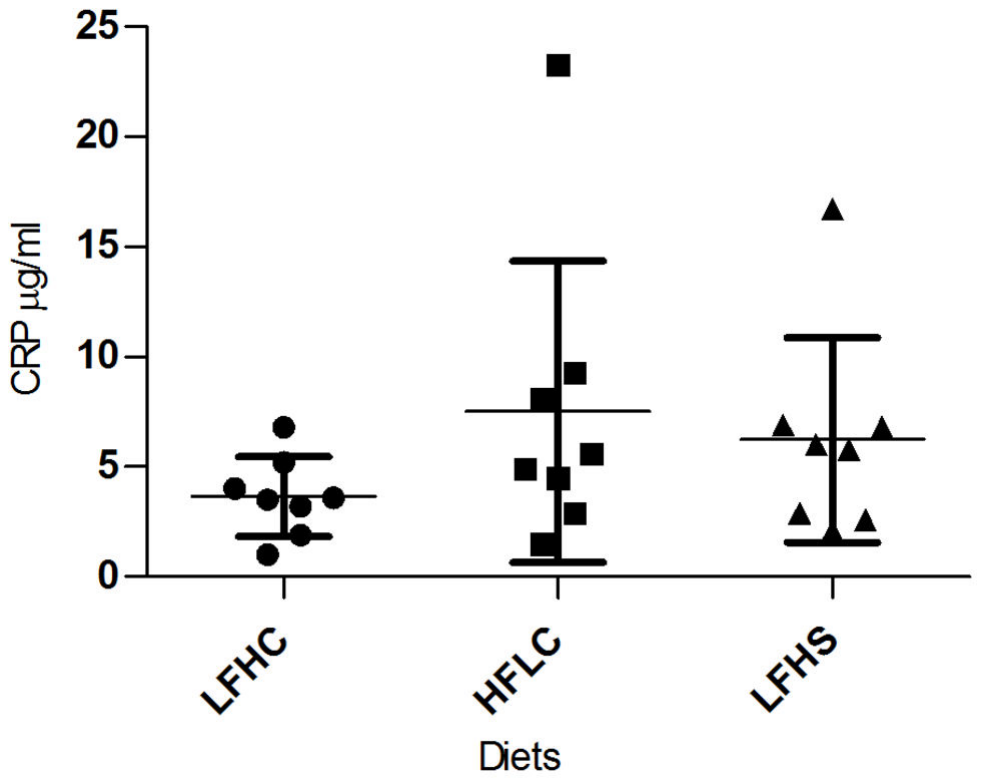
C-reactive protein (CRP) in minipig serum by the end of the study. CRP concentrations (µg/ml) ±SD for minipigs fed low fat, high carbohydrate diet (LFHC); high fat/cholesterol, low carbohydrate diet (HFLC); low fat, high carbohydrate/sucrose diet (LFHS).

**Figure 5:**
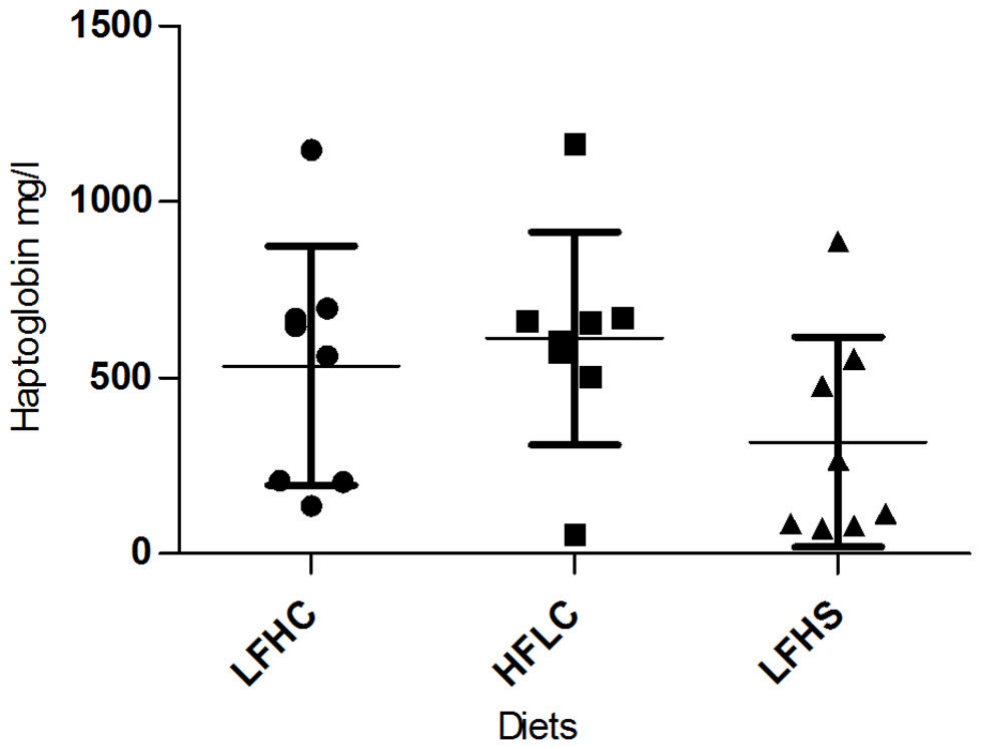
Haptoglobin in minipig serum by the end of the study. Haptoglobin concentrations (mg/l) ±SD for minipigs fed low fat, high carbohydrate diet (LFHC); high fat/cholesterol, low carbohydrate diet (HFLC); low fat, high carbohydrate/sucrose diet (LFHS).

No difference in BDNF levels between diets were found in tissue samples of frontal cortex or hippocampus (Figure 3). BDNF levels in EDTA-plasma were not detectable. Likewise, no differences between diets in concentrations of serum CRP (Figure 4) and haptoglobin (Figure 5) were found. 

**Figure 6:**
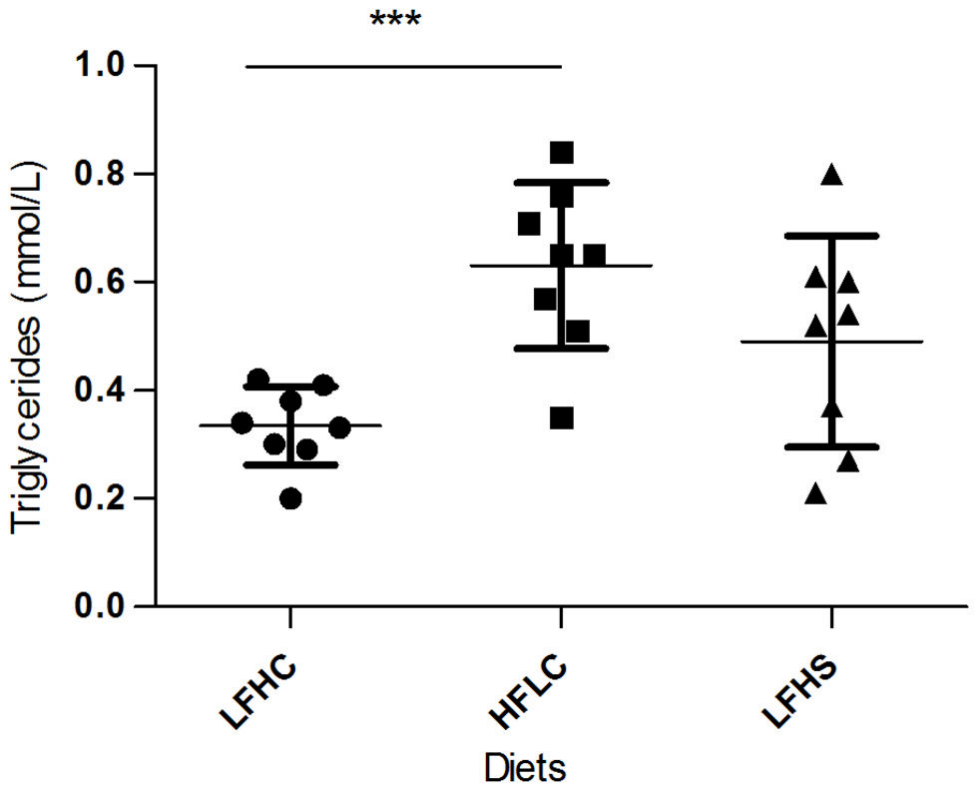
Triglycerides in minipig serum by the end of the study. Triglyceride concentrations (mmol/l) ±SD for minipigs fed low fat, high carbohydrate diet (LFHC); high fat/cholesterol, low carbohydrate diet (HFLC); low fat, high carbohydrate/sucrose diet (LFHS). *** p < 0.001).

TG levels at the end of the experiment (Figure 6) were significantly higher in HFLC minipigs compared to LFHC minipigs (t_14_ = 4.945, p < 0.001). Tendencies towards higher TG levels was found in LFHS minipigs as compared to LFHC minipigs (t_14_ = 2.122, p = 0.0522). No difference was found between LFHS- and HFLC minipigs.

**Figure 7:**
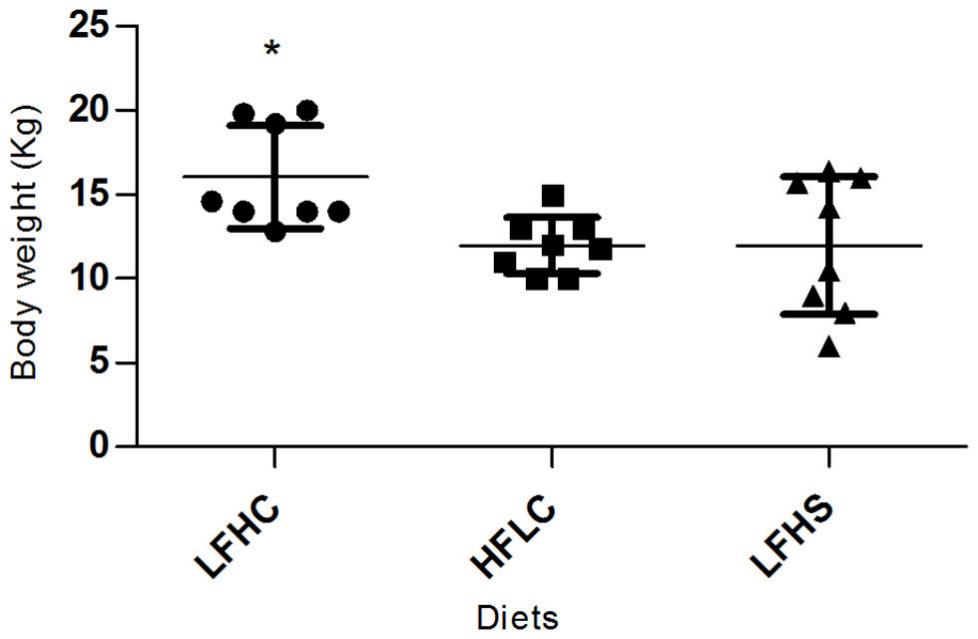
Body weight (kg) of minipigs by the end of the study. Low fat, high carbohydrate (LFHC); high fat/cholesterol, low carbohydrate (HFLC); low fat, high carbohydrate/sucrose (LFHS). * p < 0.05.

Body weights (Figure 7) differed between diet groups (LFHC vs. HFLC and LFHS) by the end of the study (F_2,21_ = 4.603, p = 0.0220), with a high variation within LFHC minipigs and LFHS minipigs. A positive correlation between body weight and trial duration was found for trial block A (13-16): (r = 0.5502, p = 0.0080, 95% CI = 0.1543 - 0.7938) and for working memory for trial block R (1-4): (r = 0.4578, p = 0.0322, 95% CI = 0.03140 - 0.7432).

## Discussion

Our study showed impaired spatial cognition of young male Göttingen minipigs performing in a spatial hole-board discrimination test after receiving a high fat/cholesterol, low carbohydrate diet or a low fat, high carbohydrate/sucrose diet. Although these two dietary treatments resulted in different performances in the spatial hole-board discrimination test, both diets seemed to have an impairing effect on adaptability to change compared with the standard minipig diet. This was primarily related to an impairment of the working memory during the reversal learning phase. Moreover, HFLC and LFHS minipigs showed retarded improvement of retention memory related to reference memory performance during the memory phase. We cannot, however, rule out the possibility that the effect on HFLC minipigs seen in our study might be due to an effect of the high saturated fat being combined with a low carbohydrate level. As both HFLC and LFHS minipigs received an increased daily amount of energy in the form of fat/cholesterol and sucrose, respectively, we are not able to conclude on a specific effect of fat/cholesterol or sucrose on cognitive performance of the minipigs. However, overall performances in the spatial hole-board discrimination test, was not influenced by body weight, supporting a possible direct effect of fat/cholesterol and sucrose. 

HFLC minipigs showed delayed improvement of reference memory acquisition compared to LFHC and LFHS minipigs (Table 2) as they significantly improved their performance only when reaching the last trial block; A(17-20). Also continuous learning during the M phase was impaired in HFLC and LFHS minipigs, as these took longer time to improve reference memory performance, remembering which bowls were baited, compared to LFHC minipigs. Furthermore, HFLC minipigs started out being significantly impaired in their working memory performance when shifted from the M phase to the R phase. Thus, they made more visits to baited bowls already visited. This supports a recent study on young men who showed a deterioration of power of attention (a measure of focused concentration) and speed of memory (representing the speed of retrieval of information from working- and episodic memory) in subjects who consumed a high fat diet for 5 days only [44]. However, as a significant positive correlation was found between working memory and body weight, for the first trial block of the R phase, it is possible that part of this difference in working memory performance between LFHC and HFLC minipigs can be explained by a difference in body weight. 

Also LFHS minipigs were compromised in continuous learning showing impaired reference memory performance compared to LFHC minipigs during the M phase. During the R phase, LFHC and HFLC minipigs were able to learn to reverse their initial acquisition of the test, both improving their working memory scores and reference memory scores between the two trial blocks. The LFHS diet impaired working memory, as these minipigs performed significantly worse than LFHC minipigs by the end of the study. These results further indicate a spatial memory impairment which might be related to the additional intake of sucrose. Recently, a study in sucrose fed male mice revealed metabolic alterations associated to type 2 diabetes and these alterations caused development of AD-like pathology [45]. However, published studies investigating cognitive effects of sugar consumption with humans are scarce. One study from 1994 reported no effects on the behaviour and cognitive performance of children, age 3-5 and 6-10 years, consuming diets high in sucrose, saccharin or aspartame [18]. In contrast, a more recent cross-sectional study on Tehrani schoolchildren, aged 6-7 years, found an inverse relationship between consumption of refined carbohydrate and non-verbal intelligence, also when adjusted for potential confounders including BMI. Non-verbal intelligence was determined by Raven’s Standard Progressive Matrices test which, amongst other things, measures the ability to organize spatial perceptions, think clearly, make sense of complexity and store and reproduce information [19]. In rodent studies, the evidence of a negative effect of sugar intake on cognition is more profound and seems to support our data from minipigs. Agrawal and Gomez-Pinilla [16] found memory impairment in fructose-fed male rats tested in a retention memory test of a Barnes maze. Similarly, Ross and colleagues showed impaired memory retention in fructose fed male rats in a spatial water maze [15]. In an attempt to explain the negative effects on cognition mechanistically, prolonged consumption of refined sugars (glucose, sucrose and fructose) was reported to decrease hippocampal neurogenesis. Here, it was found that male rats presented sugar solutions of sucrose or fructose had reduced neurogenesis, as well as increased apoptosis in the hippocampus. Interestingly the same effects were not observed for glucose-fed rats [46]. These latter studies strongly indicate that fructose consumption might play a crucial part in impairing cognitive function and, hence, also could be one cause of poor impaired performance in our minipig study, sucrose being composed partly of fructose. It should, however, be mentioned that Bruggeman and colleagues did not find spatial impairments in female fructose-fed rats, which according to the authors, indicates that the metabolism of fructose might be sex-dependent [47]. In our study, LFHS minipigs received small pieces of cube sugar as rewards when performing in the spatial hole-board discrimination test. This type of reward may potentially have blunted the impairing effect observed from the chronic supplemented sucrose, as it has been shown that sugar (glucose) consumption concomitant with a cognitive task may facilitate attention and memory in humans [48]. 

During the acquisition phase, only trial duration showed a significant effect of diet during the first trial block where LFHS minipigs took a longer time to complete a trial as compared to HFLC minipigs. Moreover, two minipigs from the LFHS diet did not habituate to the arena. It could be that minipigs fed the HFLC diet initially were more motivated or less fearful. This behaviour supports the results from a Novel object test performed on these minipigs (data not shown) where HFLC minipigs were less fearful of a novel object. 

In contrast to expectations, we found no difference in BDNF levels with relation to diet in tissue samples from hippocampus or frontal cortex. Reduced levels of BDNF in these brain regions related to feeding of a high energy diet are demonstrated in rodent studies [6,11,49-51]. One explanation could be that the minipigs in our study were all fed a restricted amount of feed every day to comply with their normal weight curve according to their age. Restricted feeding has been shown to prevent the otherwise induced decrease of BDNF levels by *ad libitum* consumption of a high energy diet, which generally is applied in rodent studies [52]. Another possible explanation involves the positive effect of physical exercise on BDNF levels; exercise being able to up-regulate BDNF [53] which according to Molteni and colleagues [22] is an effect strong enough to reverse a decrease in BDNF as well as the negative effect on cognition resulting from consumption of a high fat diet. The relatively large housing facility of the minipigs allowed for a variety of daily physical activity and when performing in the spatial hole-board discrimination test minipigs were subjected to additional physical activity twice a day. Finally, BDNF levels in the hippocampus are known to be up-regulated by learning [54], and the relatively long period of pre-exposure and testing in the spatial hole-board discrimination set-up might have contributed to an increase in BDNF levels, diminishing a possible difference between diet groups. We were unable to detect BDNF levels in plasma samples obtained before and after the introduction of diets and training. We would here have expected differences in BDNF levels related to diet. A previous study reported that plasma BDNF levels are correlated to hippocampal BDNF levels in pigs [42], and in humans, plasma BDNF levels have recently been reported to decrease as a result of consumption of a high-fat meal [51]. 

Neither did our results show any differences in serum CRP or haptoglobin between the three dietary treatments, which makes it highly unlikely that the observed impaired cognition in our study was a result of a systemic inflammatory state in the minipigs receiving the HFLC diet and the LFHS diet. 

The observed cognitive impairment in minipigs fed the HFLC and LFHS diets could thus indicate that other pathways, beside those involved in regulation of BDNF and circulatory inflammation might be responsible for the observed effects. A possible explanation could be found in the higher TG levels of HFLC and LFHS minipigs compared to LFHC minipigs. TG may alter CNS function through their breakdown into free fatty acids. An increase in circulating free fatty acids has been linked previously to reduced cognition in male human subjects [44]. However, high levels of TG might negatively affect cognitive function by other mechanisms, one of which could be through hippocampal insulin resistance [15], and another by decreasing the ability of leptin to cross the blood-brain-barrier resulting in low leptin levels, which might diminish cognitive function [55] or by impairing the NMDA (N-methyl-D-aspartate) component of hippocampal long-term potentiation [28]. 

In conclusion, feeding high energy diets of high fat/cholesterol, low carbohydrate or low fat, high carbohydrate/sucrose impairs selected domains of spatial cognition in young male Göttingen minipigs related to both working memory and reference memory. These cognitive impairments were not accompanied by decreased levels of BDNF in brain tissue and neither did they seem to be caused by circulatory inflammation. Higher levels of TG were observed for minipigs fed the diets high in fat or sugar as compared to minipigs fed a standard minipig diet. This may partially explain the impairments in spatial cognition observed in these minipigs. Our findings suggest that both increased energy intake of dietary fat and sugar might have some impairing effects on spatial cognition which could have implications for humans as well. This could be especially relevant to children and youngsters who are constantly faced with new cognitive challenges regarding learning and memory that might be more difficult to overcome due to high intake of fat and sugar. 
